# Antioxidant activity and metabolic regulation of sodium salicylate on goat sperm at low temperature

**DOI:** 10.5713/ab.23.0329

**Published:** 2024-01-20

**Authors:** Wenzheng Shen, Yu Fu, Haiyu Bai, Zhiyu Zhang, Zhikun Cao, Zibo Liu, Chao Yang, Shixin Sun, Lei Wang, Chunhuan Ren, Yinghui Ling, Zijun Zhang, Hongguo Cao

**Affiliations:** 1 College of Animal Science and Technology, Anhui Agricultural University, Hefei 230036, China; 2 Anhui Province Key Laboratory of Local Livestock and Poultry Genetic Resource Conservation and Bio-breeding, Anhui Agricultural University, Hefei, 230036, China

**Keywords:** Cryopreservation, Goat, Metabolism, Sodium Salicylate, Sperm Motility

## Abstract

**Objective:**

The purpose of this study was to explore the effect of sodium salicylate (SS) on semen preservation and metabolic regulation in goats.

**Methods:**

Under the condition of low temperature, SS was added to goat semen diluent to detect goat sperm motility, plasma membrane, acrosome, antioxidant capacity, mitochondrial membrane potential (MMP) and metabonomics.

**Results:**

The results show that at the 8th day of low-temperature storage, the sperm motility of the 20 μM SS group was 66.64%, and the integrity rates of the plasma membrane and acrosome were both above 60%, significantly higher than those of the other groups. The activities of catalase and superoxide dismutase in the sperm of the 20 μM SS group were significantly higher than those of the control group, the contents of reactive oxygen species and malondialdehyde were significantly lower than those in the control group, the MMP was significantly higher than that in the control group, and the contents of Ca^2+^ and total cholesterol were significantly higher than those in the control group. Through metabonomics analysis, there were significant metabolic differences between the control group and the 20 μM SS group. Twenty of the most significant metabolic markers were screened, mainly involving five metabolic pathways, of which nicotinic acid and nicotinamide metabolic pathways were the most significant.

**Conclusion:**

The results indicate that SS can effectively improve the low-temperature preservation quality of goat sperm.

## INTRODUCTION

Artificial insemination (AI) is the most widely used modern assisted reproductive technology (ART), which has greatly promoted the large-scale and intensive development of modern animal husbandry. The preservation of livestock semen is a major innovation of AI technology. The combination of semen preservation and AI technology can break through the time and regional restrictions on breeding livestock, and has the functions of accelerating the process of genetic improvement of livestock, protecting livestock germplasm resources and genetic diversity [1]. At present, with the improvement of living standards, the demand for mutton has rapidly increased. As a modern biotechnology, efficient preservation technology of mutton goat semen has greatly promoted the large-scale and intensive development of mutton goat breeding. At present, satisfactory results have been achieved in the preservation of semen from livestock such as cattle and horses. Due to the metabolic characteristics of goat sperm and the high requirements for preservation conditions, there are still many problems with goat semen preservation [2]. Therefore, research on the effective composition of sperm preservation solution is very important for semen preservation. For example, adding some reagents or drugs, such as oligomeric proanthocyanidin [3], kojic acid [4], salvianolic acid A [5], to the sperm diluent will significantly affect sperm motility and in vitro preservation time.

Sodium salicylate (SS) is a salicylic acid drug with anti-pyretic, analgesic, anti-inflammatory and other effects [6], which is commonly used to relieve pain and treat acute rheumatoid or rheumatoid arthritis [7]. Studies have shown that SS can regulate the morphology, structure and function of cell plasma membrane [8]. The structure of sperm is simple, and its surface is covered by a plasma membrane. The plasma membrane is easily affected by environmental conditions during sperm preservation, thereby affecting sperm motility and preservation time [9]. At present, there is limited research on the role and metabolic mechanism of SS in goat semen preservation. AMP activated protein kinase (AMPK) is a complex composed of catalytic subunits α, regulatory subunit β and γ, plays a crucial role in the regulation of cellular energy homeostasis [10]. In recent years, some studies have shown that AMPK is an important signaling pathway that regulates sperm function. AMPK can inhibit the ATP consumption pathway, accelerate the ATP synthesis pathway, maintain sperm mitochondrial membrane potential (MMP) and plasma membrane integrity, and regulate sperm motility [11,12]. Previous studies have shown that salicylates significantly increase AMPK kinase activity by increasing phosphorylation at the Thr172 site [13]. In this study, we used AMPK signaling pathway activator SS and AMPK signaling pathway inhibitor compound C (CC) [14] to investigate the role and regulatory mechanism of SS in goat semen preservation. During the low-temperature preservation of goat semen, SS can significantly improve sperm motility and prolong sperm preservation time, and regulate sperm function through mitochondrial metabolism. This research result will provide an important theoretical foundation for the application of SS in goat semen or other animal semen preservation.

## MATERIALS AND METHODS

All animal procedures were approved by the animal health care committee of Anhui Agricultural University and performed according to the legal and ethical standards in the study (SYXK2021-009).

### Animals and experimental design

Ten healthy adult male Chinese native meat goat breeds (Anhui white goats) were selected for the experiment. During the process of collecting semen, breeding rams were kept separately and freely fed.

### Collection and processing of semen

In this experiment, an artificial vagina was used to collect goat semen, and sperm motility analysis was conducted on the collected semen. Six goat semen with sperm motility higher than 80% were mixed, and the mixed semen was diluted eight times. The diluted semen was stored at a constant temperature of 4°C and used for detection and analysis.

The components of semen diluent were as follows: SS 0.16 to 0.8 mg or CC 0.04 to 0.80 mg, vitamin E 4 mL, penicillin sodium 15,000 IU, streptomycin sulfate15,000 IU, which were all bought from Shanghai yuanye Bio-Technology Co., Ltd (Shanghai, China). Fructose 1.26 g and citric acid 1.72 g, which were all bought from Shanghai Macklin Biochemical Co., Ltd (Shanghai, China). Trimethyl aminomethane 3.53 g, vitamin C 0.5 g, bovine serum albumin 0.5 g, which were all bought from Beijing Solarbio Science & Technology Co., Ltd (Beijing, China). Fresh egg yolk 20 mL, double-distilled water up to 100 mL. Diluent components are classified as analytical or guaranteed grade reagents.

### Sperm motility

A 3 μL sperm sample was placed on a preheated slide, covered with a cover glass, and analyzed for sperm motility using the CASA system (MDO2108; SCA, Mailang, China) at 37°C. Under a microscope (200×) for each sample, five fields of view were randomly selected for detection and analysis. A minimum of three replicates was performed on all data, resulting in the average of the duplicate data.

### Plasma membrane integrity

A 20 μL semen sample was placed in a 1.5 mL centrifuge tube, and 200 μL of preheated hypotonic solution was added and thoroughly mixed. Incubated at 37°C for 30 minutes, 10 μL of sperm suspension was dropped onto a slide, covered with a cover glass and air dried. The morphology of the sperm tail was examined under a microscope, and at least 200 sperm were randomly counted from five different regions of each sperm sample [15]. The curvature of the sperm tail indicates intact plasma membrane, while the straightness of the sperm tail indicates damaged plasma membrane. The percentage of intact sperm plasma membrane was calculated.

### Acrosome integrity

The Giemsa staining method was used for sperm acrosome integrity detection [16]. A 20 μL semen sample smear was air-dried, fixed with 4% formaldehyde (Hushi, Shanghai, China) for 10 to 15 minutes, rinsed and air-dried, followed by Giemsa (Macklin, Shanghai, China) staining for 1.5 to 2 hours, rinsed and air-dried. Six regions were randomly selected under a microscope, with at least 200 sperm in each region, and sperm acrosome staining was recorded. Uniform staining indicates intact sperm acrosome, while uneven staining indicates abnormal sperm acrosome. The percentage of intact sperm acrosome was calculated.

Fluorescent isothiocyanate labeled peanut agglutinin (FITC-PNA; Sigma-Aldrich, Beijing, China) staining was also used to detect sperm acrosome integrity [17]. A 20 μL semen sample was placed in a 0.5 mL centrifuge tube, 200 μL phosphate-buffered saline (PBS) solution was added, and centrifuged at 1,800 rpm for 3 minutes. The supernatant was discarded, and the precipitated sperm was fixed with 4% formaldehyde for 15 minutes. The supernatant was removed by centrifugation, and 50 μL PBS solution and 5 μL FITC-PNA staining solution were added to prepare the sperm sample for observation under a fluorescence microscope.

### Antioxidant performance

The antioxidant performance of semen samples was tested and analyzed according to the instructions of catalase (CAT) test kit (BC 0205) (Solarbio Company, Beijing, China), total antioxidant capacity (T-AOC) test kit (S0121) (Beyotime Biotechnology, Shanghai, China), and superoxide dismutase (SOD) test kit (BC 0175) (Solarbio Company, China). A 20 μL semen sample was added to 1 mL of extract, the sperm sample was ground with a grinder for 5 minutes, and the sperm supernatant was collected by centrifugation at 4°C. The sperm supernatant was placed on ice for testing, and the working solution was prepared according to the operating steps of the reagent kit. The sperm supernatant was thoroughly mixed with the working solution, and then tested using a spectrophotometer or enzyme-linked immunosorbent assay [18].

### Reactive oxygen species and malonaldehyde level

The reactive oxygen species (ROS) detection kit (S0033S) (Beyotime Biotechnology, China) was used for the detection and analysis of sperm ROS levels. DCFH-DA (fluorescent probe) was diluted with PBS solution at 1:1,000 to a concentration of 10 μM. A 20 μL semen sample was mixed with mL of DCFH-DA solution and incubated at 37°C for 20 minutes. Sperm was washed with PBS solution and resuspended. Enzyme linked immunosorbent assay was used to measure absorbance at excitation wavelength 488 nm and emission wavelength 525 nm [19].

The malonaldehyde (MDA) detection kit (BC 0025) (Solarbio Company, China) was used to detect the MDA level of semen samples. A 20 μL semen sample was added to 1 mL of extraction solution, ground with a grinder for 5 minutes, centrifuged at 4°C for 10 minutes, and the sperm supernatant was collected. The working solution was prepared according to the requirements of the kit, and the supernatant was thoroughly mixed with the working solution. The absorbance of the sample at 532 nm and 600 nm was measured using enzyme-linked immunosorbent assay.

### Mitochondrial membrane potential and ATP level

The detection of MMP was carried out according to the instructions of the MMP detection kit (C2006) (Beyotime Biotechnology, China). Flow cytometry and fluorescence microscopy were used for detection and analysis. The collected semen samples were centrifuged at 1,800 rpm for 3 minutes, and the supernatant was removed. The concentration of sperm was adjusted to 1×10^6^ cells/mL using PBS solution. A 100 μL of semen sample was placed in a 1.5 mL centrifuge tube, followed by 0.5 mL of JC-1 (fluorescent probe for detecting MMP) staining solution, and incubated at 37°C for 20 minutes. The supernatant was removed by centrifugation. The precipitated sperm was resuspended with JC-1 staining buffer (1×) and then detected by flow cytometry [20]. In addition, the sperm sample stained with 20 μL JC-1 staining solution was placed on a slide and covered with a cover glass for detection and analysis under a fluorescence microscope. The relative ratio of red and green fluorescence is often used to measure the degree of mitochondrial depolarization.

According to the instructions of the ATP detection kit (S0026) (Beyotime Biotechnology, China), the ATP content in sperm was detected and analyzed. A 20 μL semen sample was placed in 1 mL of cell lysate, centrifuged at 4°C for 10 minutes, and the sperm supernatant was collected. The working fluid was prepared according to the requirements of the kit, and the sperm supernatant was added to the working fluid, thoroughly mixed, and then the sperm ATP content was detected and analyzed using enzyme-linked immunosorbent assay. The ATP content in the semen samples was calculated from an ATP standard curve, and the results were expressed in units of nmol/mg [21].

## Ca^2+^ and TC content

The Ca^2+^ content in sperm was detected using a Ca^2+^ content detection kit (R22060) (Yuanye Biotechnology, Shanghai, China). A 20 μL semen sample was added to 1 mL of extraction solution, ground with a grinder for 5 minutes, centrifuged at 4°C for 10 minutes, and the supernatant was collected and placed on ice. The working solution was prepared according to the requirements of the kit. The working solution was thoroughly mixed with the sample supernatant, and the absorbance of the sample at 610 nm was measured using enzyme-linked immunosorbent assay, all operating steps and calculation methods were strictly carried out in accordance with the instructions of the reagent kit [22].

The total cholesterol (TC) content in semen samples was detected using the TC detection kit (BC 1980) (Solarbio Company, China). A 20 μL semen sample was added to 1 mL of extraction solution, ground with a grinder for 5 minutes, centrifuged at 4°C for 10 minutes, and the supernatant was collected and placed on ice. The working solution was prepared according to the requirements of the kit, and the absorbance of the sample at 500 nm was measured using a spectrophotometer. All operating steps were carried out according to the instructions of the reagent kit [23].

### Metabolomics analysis

For the control group and SS group of goat sperm, nontargeted metabolomics analysis was performed on each group of samples (n = 8). Briefly, 1,000 μL of isotope labeled extract was added to the sperm sample, mixed and rotated for 30 seconds. The sample was freeze-thawed 3 times in liquid nitrogen, sonicated in an ice water bath for 10 minutes, incubated at −40°C for 1 hour, centrifuged at 12,000 rpm at 4°C for 15 minutes, and the supernatant was collected for LC/MS analysis, LC-MS/MS analysis was performed using the UHPLC system (Vanquish, Thermo Fisher Scientific, Waltham, MA, USA) [24,25].

### Statistical analysis

The data was analyzed using SPSS software, and changes in sperm motility, plasma membrane integrity rate, and acrosome integrity rate were analyzed using one-way analysis of variance. Tukey test was used for multiple comparisons, and the results were presented in the form of mean±standard deviation. T-test was used to compare the differences in antioxidant capacity, MMP, ATP, Ca^2+^, and TC between the control group and the optimal concentration of 20 μM SS group, with p<0.05 indicating significant differences, and p<0.01 indicating significant difference. GraphPad Prism 8 was used to plot the data, and each treatment must have at least three replicates.

## RESULTS

### Effects of SS on sperm quality

The impact of SS on sperm quality is shown in Table 1. Under the condition of 4°C, the sperm motility of goats gradually decreased with the prolongation of storage time, but the results showed that SS had a good protective effect on goat sperm, with 20 μM SS having the best protective effect. Starting from the 2nd day, the motility of the 20 μM SS group was significantly higher than that of the other groups (p<0.05), and after 8 days of storage, the sperm motility still reached over 60%. Starting from the 3rd day, the 30 μM SS group was significantly higher than the control group (p<0.05). There was no significant difference in sperm motility between the 10 μM or 50 μM SS groups and the control group (p>0.05).

**Figure 1:**
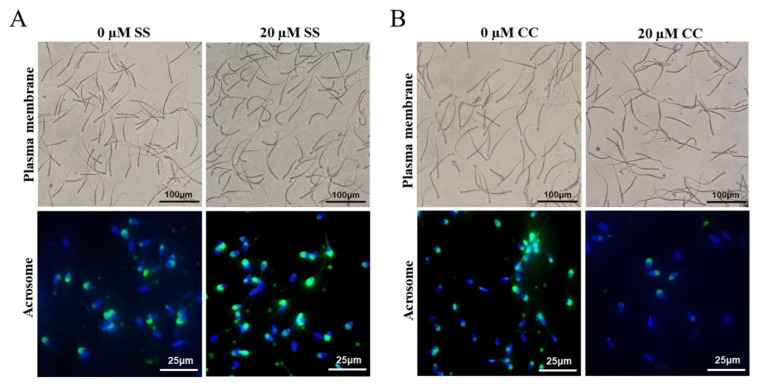
Detection of the effect of sodium salicylate and compound C on the integrity of sperm plasma membrane and acrosome. (A) The effect of sodium salicylate (SS) on the integrity of sperm plasma membrane and acrosome (Day 6). (B) The effect of compound C (CC) on the integrity of sperm plasma membrane and acrosome (Day 4). The curvature of the sperm tail indicates intact plasma membrane, while the straightness of the sperm tail indicates damaged plasma membrane. Green fluorescence represents sperm acrosome staining, while blue fluorescence represents sperm DNA staining.

At 4°C, SS had a protective effect on goat sperm plasma membrane, and the sperm plasma membrane integrity rate showed a characteristic of first increasing and then decreasing with the increase of SS concentration. Among the SS groups with different concentrations, the 20 μM SS group had the best sperm plasma membrane integrity rate. Starting from day 3, the sperm plasma membrane integrity rate of the 20 μM SS group was significantly higher than that of the other groups (p<0.05) (Figure 1A). In addition, the sperm plasma membrane integrity rate of the 10 μM or 30 μM SS group was significantly higher than that of the control group (p< 0.05), and there was no significant difference between the 50 μM SS and control group (p>0.05).

The trend of acrosome integrity rate was similar to that of plasma membrane integrity rate, the 20 μM SS group was significantly higher than other groups from the 3rd day of storage (p<0.05). There was no significant difference between the 10 μM and 30 μM SS groups (p>0.05), and most of them were significantly higher than the control group (p<0.05). There was no significant difference between the 50 μM SS group and the control group (p>0.05). The results showed that the 20 μM SS group could significantly improve the sperm acrosome integrity rate.

### Effects of CC on sperm quality

The impact of CC on sperm quality is shown in Table 2. After adding 1, 5, 10, and 20 μM CC to the goat sperm preservation solution, the sperm motility of the goats showed varying degrees of decrease. On the 2nd day of storage, the sperm motility of the 20 μM CC group was significantly lower than that of the control group (p<0.05); the sperm motility of the 5 μM and 10 μM CC group was significantly lower than that of the control group starting from the 4th day of sperm preservation (p<0.05); on the 5th day of sperm preservation, the sperm motility of the 1 μM CC group was significantly lower than that of the control group, and the sperm motility of the 20 μM CC group was significantly lower than that of other groups (p<0.05).

The integrity rate of goat sperm plasma membrane showed a decreasing trend with the increase of CC concentration. Starting from the 3rd day, the sperm plasma membrane integrity rate in the 10 μM and 20 μM CC group was significantly lower than that in the control group (p<0.05). From the 4th day, the sperm plasma membrane integrity rate in the 20 μM CC group was significantly lower than that in the 10 μM CC group (p<0.05) (Figure 1B). The sperm plasma membrane integrity rate in the 5 μM CC group was significantly lower than that in the control group on the 4th day (p<0.05).

The acrosome integrity rate of goat sperm gradually decreased with the increase of CC concentration. When sperm were stored for 3 days, the acrosome integrity rate of each treatment group was significantly lower than that of the control group (p<0.05). When saving to 4 d, the sperm acrosome integrity rate in the 20 μM CC group was significantly lower than that in other treatment groups (p<0.05).

### Analysis of SS and CC on antioxidant ability of goat sperm

Through the detection of goat sperm quality, it was found that the sperm quality showed a characteristic of first increasing and then decreasing with the increase of SS concentration, and the optimal concentration of SS was 20 μM. The quality of sperm gradually decreased with the increase of CC concentration, and 20 μM CC had serious damage to the quality of sperm, therefore, used 20 μM SS and 20 μM CC when conducting antioxidant index testing.

**Figure 2:**
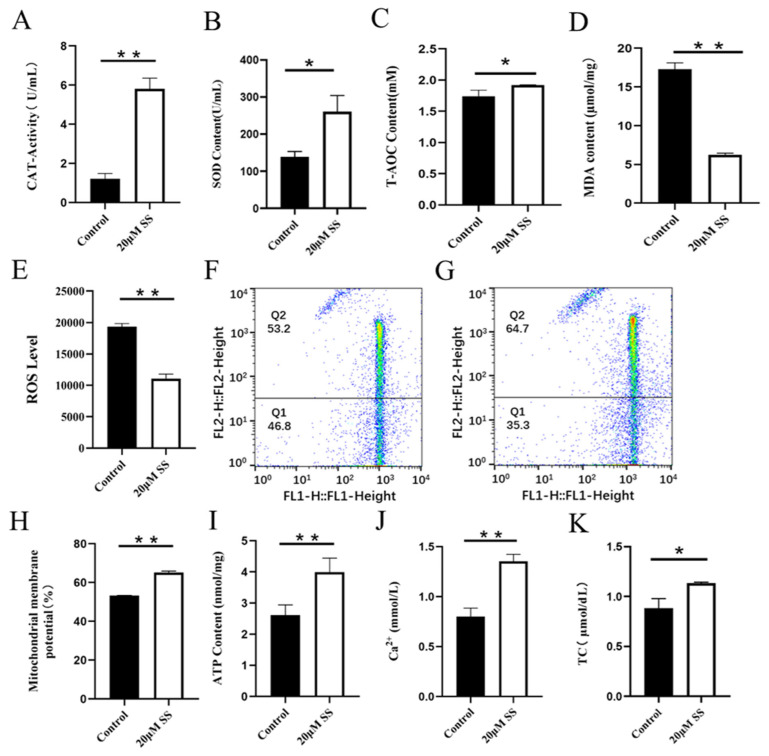
Detection of sodium salicylate on sperm antioxidant capacity, mitochondrial energy metabolism and TC (Day 6). (A) The effect of SS on CAT activity. (B) The effect of SS on SOD activity. (C) The effect of SS on T-AOC level. (D) The effect of SS on the MDA content. (E) The effect of SS on the ROS level. (F) MMP level in control group by flow cytometry (G) MMP level in SS group by flow cytometry (H) The effect of SS on MMP level. (I) The effect of SS on ATP content. (J) The effect of SS on Ca^2+^ level. (K) The effect of SS on TC level. * p<0.05, ** p<0.01. TC, total cholesterol; SS, sodium salicylate; CAT, catalase; SOD, superoxide dismutase; T-AOC, total antioxidant capacity; MDA, malondialdehyde; ROS, reactive oxygen species; MMP, mitochondrial membrane potential.

**Figure 3:**
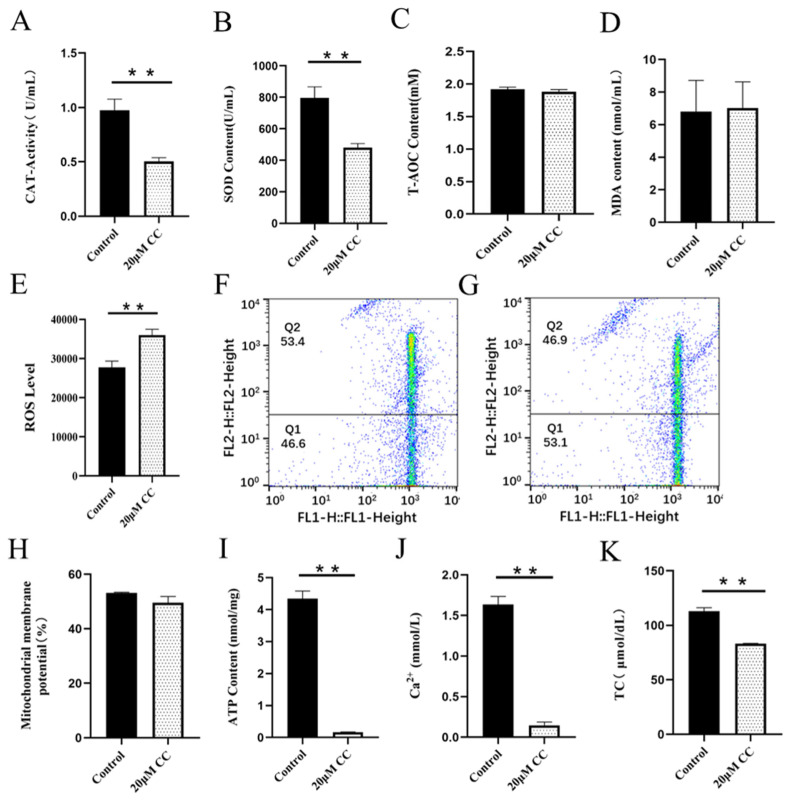
Detection of compound C on sperm antioxidant capacity, mitochondrial energy metabolism and TC (Day 4). (A) The effect of CC on CAT activity. (B) The effect of CC on SOD activity. (C) The effect of CC on T-AOC level. (D) The effect of CC on the MDA content. (E) The effect of CC on the ROS level. (F) MMP level in control group by flow cytometry (G) MMP level in CC group by flow cytometry (H) The effect of CC on MMP level. (I) The effect of CC on ATP content. (J) The effect of CC on Ca^2+^ level. (K) The effect of CC on TC level. * p<0.05, ** p<0.01. TC, total cholesterol; CC, compound C, CAT, catalase; SOD, superoxide dismutase; T-AOC, total antioxidant capacity; MDA, malondialdehyde; ROS, reactive oxygen species; MMP, mitochondrial membrane potential.

CAT is an endogenous antioxidant enzyme, which can protect cells from ROS damage. Adding SS to semen preservation solution significantly increased the activity of goat sperm CAT, which was significantly different from the control group (p<0.01; Figure 2A). The CAT activity in the control group was significantly higher than that in the CC treatment group (p<0.01; Figure 3A).

SOD is a kind of metal enzyme that can eliminate free radicals and resist oxidation in the body. The results showed that adding SS could effectively increase the SOD content of sperm, and it was significantly higher than the control group (p<0.05; Figure 2B). The detection of CC significantly reduced sperm SOD content, and the difference between the CC group and the control group was extremely significant (p<0.01; Figure 3B).

T-AOC refers to the total antioxidant level composed of various antioxidant substances and antioxidant enzymes. Adding SS to the preservation solution effectively increases sperm T-AOC, with a significant difference compared to the control group (p<0.05; Figure 2C); when detecting T-AOC in the CC treatment group, it was found that although the content of T-AOC in the CC treatment group was lower than that in the control group, the difference was not significant (p>0.05; Figure 3C).

MDA is the final product of lipid peroxidation, and its concentration can reflect the degree of lipid peroxidation, and its production can also exacerbate membrane damage. The results showed that the SS treatment group could effectively reduce the MDA level of goat sperm, and the difference was extremely significant compared to the control group (p<0.01; Figure 2D). The level of MDA in sperm of CC treatment group was higher than that of control group, but there was no statistical significance (p>0.05; Figure 3D).

ROS mainly comes from the intracellular mitochondrial respiratory chain, which is generated by mitochondria and some enzyme reactions. Adding 20 μM SS to goat sperm preservation solution significantly reduced sperm ROS levels (p<0.01; Figure 2E), 20 μM CC treatment group had significantly increased ROS content (p<0.01; Figure 3E).

### Regulatory analysis of SS and CC on MMP and ATP in goat sperm

**Figure 4:**
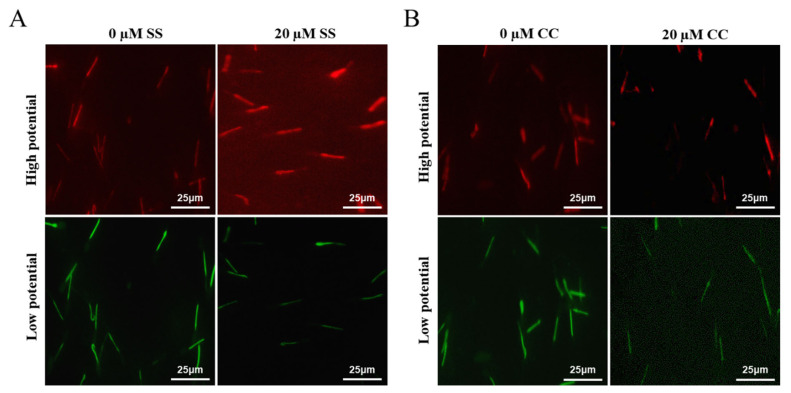
The effects of sodium salicylate and compound C on sperm MMP using JC-1. (A) The effect of SS on MMP of Sperm (Day 6). (B) The effect of CC on MMP of sperm (Day 4). Red fluorescence indicates high membrane potential staining, and green fluorescence indicates low membrane potential staining. MMP, mitochondrial membrane potential; SS, sodium salicylate; CC, compound C.

Mitochondria are key components involved in energy metabolism. MMP is one of the important parameters reflecting the functional state of mitochondria in cells. The higher the MMP, the more energy produced by mitochondria, which promotes cell energy conversion. The decline of MMP is a marker event of early apoptosis. JC-1 fluorescent probe was used to detect the MMP of goat spermatozoa. On the 6th day of semen preservation, flow cytometry analysis showed that the high potential of spermatozoa in SS group was 64.7% (Figure 2F; Figure 4A), and that in control group was 53.2% (Figure 2G). The potential of spermatozoa in SS group was significantly higher than that in control group (p<0.01). On the 5th day of semen preservation, in the CC group, the high potential was 46.9% (Figure 3F; Figure 4B), while in the control group, the high potential was 53.4% (Figure 3G). The high potential in CC group was lower than that in control group, but the difference was not significant (p>0.05). The results showed that the addition of SS could effectively improve the MMP of goat sperm, while the addition of CC significantly reduced the MMP.

ATP is the energy source for sperm motility, and ATP content is also an important reference standard for evaluating sperm mitochondrial activity. Adding SS to goat sperm preservation solution could effectively increase sperm ATP levels, which was significantly higher than that of the control group (p<0.01; Figure 2I). Meanwhile, the ATP content of the CC group was detected and the ATP content of the control group was significantly higher than that of the CC group (p<0.01; Figure 3I).

### Regulatory analysis of SS and CC on Ca^2+^ and TC in goat sperm

Adding SS to goat sperm preservation solution increased sperm Ca^2+^ content, which was significantly different from the control group (p<0.01; Figure 2J), while CC reduced sperm Ca^2+^ content (p<0.01; Figure 3J).

Cholesterol is the basic component of cell membranes, which can regulate the fluidity and permeability of cell membranes. In goat semen preservation solution, the SS group had significantly increased sperm TC levels (p<0.05; Figure 2K), while the CC group had significantly reduced sperm TC levels (p<0.01; Figure 3K).

### Regulatory characteristics of SS on sperm metabolism

**Figure 5:**
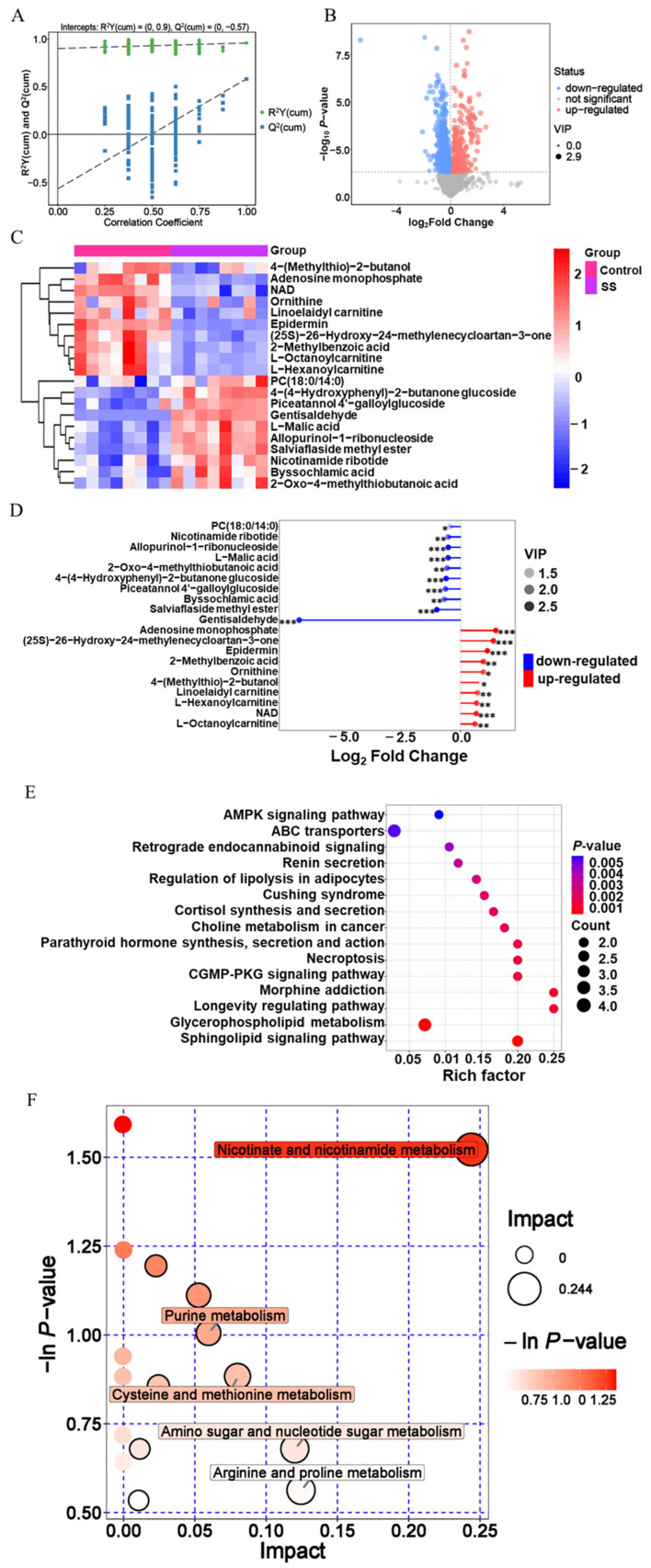
Regulation and differential metabolism of sodium salicylate on goat sperm (Day 6). (A) OPLS-DA model of metabolites. (B) Screening of differential metabolites between control group and SS group. (C) Differential metabolites of goat sperm between SS group and control group. (E) Match rod map of differential metabolites in SS group. (E) KEGG enrichment analysis of differential metabolites in SS group. (F) Analysis of the metabolic pathway of goat sperm in SS group. OPLS-DA, orthogonal partial least squares discriminant analysis; SS, sodium salicylate; KEGG, Kyoto encyclopedia of genes and genomes. Regulation and differential metabolism of sodium salicylate on goat sperm (Day 6). (A) OPLS-DA model of metabolites. (B) Screening of differential metabolites between control group and SS group. (C) Differential metabolites of goat sperm between SS group and control group. (E) Match rod map of differential metabolites in SS group. (E) KEGG enrichment analysis of differential metabolites in SS group. (F) Analysis of the metabolic pathway of goat sperm in SS group. OPLS-DA, orthogonal partial least squares discriminant analysis; SS, sodium salicylate; KEGG, Kyoto encyclopedia of genes and genomes.

Sperm samples were analyzed using non targeted metabolomics (Figure 5A; Supplementary Table S1). Through metabolomic data analysis, 4,708 metabolites were identified in goat sperm. Among them, 985 differential metabolites were detected in the SS group, with 300 upregulated and 685 downregulated (Figure 5B; Supplementary Tabe S2).

According to the differential metabolite clustering heat map analysis of goat sperm Supplementary (Supplementary Figure S1), 10 metabolites such as 4-(methylthio)-2-butanol, adenosine monophosphate, and nicotinamide adenine dinucleotide (NAD) were significantly downregulated (p<0.05); ten metabolites, including 4-(4-hydroxyphenyl)-2-butanone glucoside, PC (18:0/14:0), and L-neneneba malic acid, were significantly up-regulated (p<0.05) in the SS group (Figure 5C). In addition, gentisaldehyde, the down regulated metabolite, was the most variable metabolite. Adenosine monophosphate had the highest multiple changes, the variable importance in projection (VIP) value of the metabolite of epidermin was the highest and most significant in the SS group (Figure 5D; Table 3).

The correlation between differential metabolites was analyzed, and the correlations between metabolites during goat sperm preservation were analyzed. Adenosine monophosphate and allopurinol-1-ribonucleioside (correlation coefficient r = −0.95), salviaflaside methyl ester and NAD (r = −0.94), gentisaldehyde and adenosine monophosphate (r = −0.86) showed strong negative correlations; gentisaldehyde and allopurinol-1-ribonucleoside (r = 0.90), salviaflaside methyl ester and 4-(4-hydroxyphenyl)-2-butanone glucoside (r = 0.91), L-malic acid and salviaflaside methyl ester (r = 0.95) were strongly positively correlated (Supplementary Figure S2).

In SS group, 87 pathways were finally enriched (Supplementary Tabe S3), which were mainly involved in metabolic pathways, sphingomyelin signaling pathway, ABC transporters, etc. Among them, metabolic pathways were the most abundant, with 20 differential metabolites, the metabolic pathway of sphingomyelin signaling pathway had the most significant enrichment (p<0.05) (Figure 5E; Table 4).

In SS group, differential metabolites were involved in 17 metabolic pathways (Supplementary Table S4), of which 5 major metabolic pathways were affected, including nicotinate and nicotinamide metabolism, arginine and proline metabolism, purine metabolism, cysteine and methionine metabolism, and amino sugar and nucleotide sugar metabolism. Among them, nicotinate, and nicotinamide metabolism pathways have the greatest impact, and its p-value was also the smallest, with the most significant enrichment (p<0.05) (Figure 5F; Table 5).

**Figure 6:**
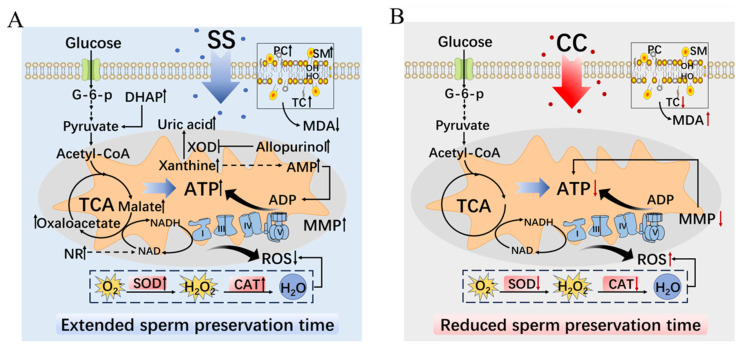
The regulatory mechanism of sodium salicylate and compound C on goat sperm metabolism. (A) The mechanism of AMPK activator SS regulating goat sperm metabolism. (B) The mechanism of AMPK inhibitor CC regulating goat sperm metabolism. SS, sodium salicylate; CC, compound C; G-6-p, glucose-6-phosphate; DHAP, dihydroxyacetone phosphate; TCA, tricarboxylic acid cycle; NR, nicotinamide riboside; PC, phosphatidylcholine; SM, sphingomyelin; TC, total cholesterol; MDA, malondialdehyde; XOD, xanthine oxidase; AMP, adenosine monophosphate; ADP, adenosine diphosphate; MMP, mitochondrial membrane potential; NADH, nicotinamide adenine dinucleotide; NAD, nicotinamide adenine dinucleotide; SOD, superoxide dismutase; CAT, catalase; ROS, reactive oxygen species.

The experimental results confirmed that SS can prolong the in vitro preservation time and quality of goat sperm by enhancing its antioxidant capacity and regulating sperm metabolism under low temperature conditions (Figure 6A). On the contrary, CC affects the in vitro preservation time and quality of goat sperm by reducing their antioxidant capacity (Figure 6B).

## RESULTS

SS, as a chemical reagent and drug, is widely used in daily life. SS has a certain analgesic effect and is a commonly used medication for treating rheumatism [26]. It is used as a food additive for its anti-corrosion effect [27]. Due to the relatively small side effects of this drug on humans, many gout patients will take it to alleviate their adverse symptoms [28]. In human clinical applications, SS serves as a nuclear factor-κB inhibitor, used to prevent sepsis caused by endotoxin lipopolysaccharide [29]. Other studies have shown that SS inhibits the release of Cu/Zn Superoxide dismutase, ROS production and cell necrosis induced by glucose depletion [30], and it can activate an energy sensor called AMPK, which maintains a balance between ATP production and consumption [31].

Hawley et al [13] have shown that SS and its derivatives regulate various mitochondrial metabolic processes by activating the AMPK signaling pathway, thereby maintaining intracellular energy balance. AMPK is an important protein that plays an important role in mammalian sperm, maintaining energy balance, and serving as a sensor for cellular energy status [32]. In the study of this signal pathway, it was found that the activation of AMPK has certain effects on various aspects of sperm, such as motility, MMP, acrosomal membrane integrity, and plasma membrane stability [33]. And it can reduce ROS content and improve the antioxidant system of sperm [34]. In this study, by adding AMPK signaling pathway inhibitor CC, the results showed that CC can significantly reduce the quality of goat sperm preservation, AMPK pathway activator SS plays a protective role on goat sperm by affecting sperm plasma membrane and metabolism.

SS protects cortical neurons by avoiding depolarization of the plasma membrane caused by acidosis [35], and can act on the lipid bilayer of the cell membrane, causing morphological changes on the membrane surface [8]. As a nonsteroidal anti-inflammatory drug, the pharmacological effect of SS comes from its interaction with lipid bilayer components of cell membranes [36]. For the structure of the cell plasma membrane, SS participates in molecular rearrangement of the cell plasma membrane through interactions between COOH terminals and COOH- and NH (2)-terminals [37]. The morphology, structure, and function of the cell membrane regulated by SS are consistent with the results of this experiment. At the same time, we also confirmed that SS can improve the integrity of sperm plasma membrane and acrosome.

During cryopreservation of goat spermatozoa, through metabonomic analysis of SS group, the most significant difference between the control group and SS group was nicotinic acid and nicotinamide metabolism, and the differential metabolite involved was NAD. Nicotinic acid turns into nicotinamide in vivo, nicotinamide is the precursor of NAD+. NAD is a key molecule of cell intermediate metabolism and participates in glycolysis, electron transport chain, oxidative phosphorylation and other processes. NAD is constantly consumed and degraded by some enzymes during cellular metabolism, and maintaining its level is crucial for ATP biosynthesis and mitochondrial homeostasis [38]. Some studies have shown that nicotinamide mononucleotide can reduce spermatogenic cell apoptosis and play its protective role by regulating the glycolysis of sertoli cells in diabetes mice [39].

Torres et al [40] studied that nicotinamide in cryopreserved sperm metabolic markers is related to energy metabolism and can improve sperm quality. In conclusion, SS may affect the sperm preservation effect through nicotinic acid and nicotinamide metabolism in goat sperm. However, most of the up-regulated differential metabolites are lipid components such as sphingomyelin and phosphatidylcholine. Sphingomyelin is an important part of cell membranes. Goat sperm in SS group may improve sperm preservation effect through sphingomyelin signal pathway.

The study of SS in semen preservation provides an important theoretical basis for the efficient utilization of ram semen and lays the foundation for the efficient utilization of reproductive performance in other breeds of male livestock.

## Figures and Tables

**Table 1:** Effects of different concentrations of sodium salicylate on the motility, plasma membrane integrity, and acrosome integrity of goat sperm preserved at 4°C

Time/d	Con (μM)	Motility (%)	Plasma membrane integrity	Acrosome integrity
1	0	80.66±2.13^ab^	0.84±0.03^a^	0.86±0.01^a^
	10	80.09±2.95^ab^	0.85±0.03^a^	0.87±0.03^a^
	20	83.69±2.23^a^	0.86±0.02^a^	0.89±0.02^a^
	30	79.50±2.45^b^	0.83±0.02^a^	0.86±0.02^a^
	50	83.05±1.63^ab^	0.84±0.03^a^	0.88±0.01^a^
2	0	77.23±0.93^b^	0.83±0.01^b^	0.84±0.01^ab^
	10	79.73±1.47^b^	0.84±0.02^ab^	0.84±0.02^ab^
	20	83.27±1.38^a^	0.85±0.02^a^	0.88±0.03^a^
	30	79.48±3.23^b^	0.82±0.01^b^	0.83±0.04^ab^
	50	79.20±1.43^b^	0.81±0.01^b^	0.80±0.02^b^
3	0	72.03±0.94^c^	0.74±0.03^d^	0.77±0.01^c^
	10	78.70±2.49^b^	0.81±0.01^b^	0.83±0.02^b^
	20	81.73±2.15^a^	0.85±0.03^a^	0.86±0.02^a^
	30	79.36±1.37^ab^	0.79±0.02^bc^	0.80±0.01^b^
	50	74.26±1.82^c^	0.76±0.03^cd^	0.78±0.01^c^
4	0	72.02±1.03^c^	0.72±0.02^c^	0.74±0.02^c^
	10	74.00±2.27^bc^	0.75±0.02^bc^	0.78±0.02^b^
	20	80.91±3.21^a^	0.82±0.02^a^	0.83±0.02^a^
	30	76.62±2.47^b^	0.77±0.02^b^	0.76±0.02^bc^
	50	72.84±0.79^c^	0.72±0.02^c^	0.74±0.03^c^
6	0	67.86±2.53^c^	0.67±0.02^cd^	0.70±0.04^b^
	10	71.29±2.68^bc^	0.70±0.03^bc^	0.73±0.01^b^
	20	79.99±2.12^a^	0.81±0.01^a^	0.83±0.02^a^
	30	74.72±0.93^b^	0.73±0.03^b^	0.74±0.02^b^
	50	68.82±2.69^c^	0.66±0.02^d^	0.70±0.01^b^
8	0	58.88±1.52^b^	0.56±0.02^d^	0.62±0.03^bc^
	10	59.27±1.05^b^	0.59±0.02^c^	0.63±0.03^b^
	20	66.64±2.04^a^	0.70±0.01^a^	0.69±0.01^a^
	30	59.20±0.68^b^	0.63±0.02^b^	0.61±0.02^bc^
	50	54.36±3.01^c^	0.56±0.01^d^	0.59±0.03^c^

Con, concentration (μM).

a–d In the same column within the same preservation time, different letters indicate significant differences (p<0.05).

**Table 2:** Effects of different concentrations of compound C on the motility, plasma membrane integrity, and acrosome integrity of goat sperm preserved at 4°C

Time/d	Con (μM)	Motility (%)	Plasma membrane integrity	Acrosome integrity
1	0	81.51±1.05^a^	0.84±0.01^a^	0.85±0.01^a^
	1	84.76±1.23^ab^	0.85±0.01^a^	0.86±0.02^a^
	5	83.67±2.78^b^	0.84±0.02^a^	0.86±0.02^a^
	10	82.29±1.84^b^	0.82±0.02^a^	0.87±0.01^a^
	20	88.30±1.69^a^	0.86±0.02^a^	0.87±0.02^a^
2	0	81.44±3.37^b^	0.84±0.03^a^	0.83±0.02^a^
	1	78.16±2.51^b^	0.84±0.04^a^	0.83±0.02^a^
	5	77.85±1.62^b^	0.82±0.04^a^	0.82±0.02^a^
	10	77.31±1.46^bc^	0.81±0.03^a^	0.82±0.03^a^
	20	74.49±1.64^c^	0.80±0.01^a^	0.80±0.01^a^
3	0	77.19±1.32^a^	0.77±0.02^a^	0.80±0.01^a^
	1	74.42±1.29^ab^	0.75±0.02^ab^	0.78±0.01^b^
	5	73.16±3.58^bc^	0.72±0.02^bc^	0.75±0.01^c^
	10	71.89±1.09^bc^	0.72±0.02^bc^	0.73±0.02^cd^
	20	70.22±0.80^c^	0.69±0.03^c^	0.71±0.02^d^
4	0	73.23±2.06^a^	0.73±0.01^a^	0.74±0.02^a^
	1	70.29±2.23^a^	0.71±0.02^ab^	0.74±0.02^a^
	5	66.50±1.70^b^	0.67±0.02^b^	0.69±0.01^b^
	10	65.59±2.44^b^	0.67±0.03^b^	0.66±0.02^b^
	20	63.31±2.60^b^	0.61±0.03^c^	0.63±0.02^c^
5	0	71.48±0.87^a^	0.70±0.02^a^	0.70±0.04^a^
	1	66.99±1.52^b^	0.65±0.03^b^	0.64±0.03^b^
	5	63.11±2.68^c^	0.61±0.01^c^	0.60±0.02^bc^
	10	60.69±2.40^c^	0.60±0.02^c^	0.58±0.01^c^
	20	45.59±2.82^d^	0.53±0.02^d^	0.52±0.03^d^

Con, concentration (μM).

a–d In the same column within the same preservation time, different letters indicate significant differences (p<0.05).

**Table 3:** Differential metabolites of goat sperm between sodium salicylate group and control group

Name	VIP	p-value^1)^	FC
PC (18:0/14:0)	1.362206210	0.043608793	0.736346476
Nicotinamide ribotide	2.102586779	0.002393376	0.699609468
Allopurinol-1-ribonucleoside	2.762349375	0.000003151	0.698590682
L-Malic acid	2.638041025	0.000042514	0.695575174
2-Oxo-4-methylthiobutanoic acid	2.158343046	0.003585855	0.674141511
4-(4-Hydroxyphenyl)-2-butanone glucoside	2.791796338	0.000004535	0.655972813
Piceatannol 4′-galloylglucoside	2.325467613	0.000593739	0.643175094
Byssochlamic acid	2.059025952	0.003565775	0.621742163
Salviaflaside methyl ester	2.833766695	0.000000386	0.498737361
Gentisaldehyde	2.638450262	0.000000005	0.008721041
Adenosine monophosphate	2.804301062	0.000118680	2.822727448
(25S)-26-Hydroxy-24-methylenecycloartan-3-one	2.676963330	0.000081262	2.634815921
Epidermin	2.908850948	0.000000375	2.026151414
2-Methylbenzoic acid	2.488806063	0.001188453	1.965472492
Ornithine	2.131267615	0.021554356	1.960443355
4-(Methylthio)-2-butanol	1.074108892	0.049769743	1.719759251
Linoelaidyl carnitine	2.137360075	0.006044277	1.661416221
L-Hexanoylcarnitine	2.430268454	0.001959969	1.607006542
NAD	2.526394442	0.000033699	1.594467573
L-Octanoylcarnitine	2.388624059	0.003719808	1.538046502

VIP, variable importance in projection; FC, fold change; PC, phosphatidylcholine; NAD, nicotinamide adenine dinucleotide.

1) p-value: the p-value obtained from the t-test of the substance in this group comparison.

**Table 4:** Enrichment analysis of differential metabolites in goat sperm from sodium salicylate group

Name	p-value^1)^	Number of differential metabolites
AMPK signaling pathway	0.005847929	2
ABC transporters	0.005461604	4
Retrograde endocannabinoid signaling	0.004371097	2
Renin secretion	0.003498951	2
Regulation of lipolysis in adipocytes	0.002364012	2
Cushing syndrome	0.002032856	2
Cortisol synthesis and secretion	0.001725679	2
Choline metabolism in cancer	0.001442724	2
Parathyroid hormone synthesis, secretion and action	0.001184235	2
Necroptosis	0.001184235	2
cGMP-PKG signaling pathway	0.001184235	2
Morphine addiction	0.000741641	2
Longevity regulating pathway	0.000741641	2
Glycerophospholipid metabolism	0.000185296	4
Sphingolipid signaling pathway	0.000057204	3

1) p-value: the p-value obtained from the t-test of the substance in this group comparison.

**Table 5:** Analysis of the metabolic pathway of goat sperm in sodium salicylate group

Pathway	Total^1)^	Hits^2)^	Raw p^3)^	Holm adjust^4)^	Impact^5)^
Nicotinate and nicotinamide metabolism	13	1	0.21836	1	0.2439
Arginine and proline metabolism	44	1	0.56977	1	0.12445
Purine metabolism	68	2	0.36603	1	0.05954
Cysteine and methionine metabolism	28	1	0.41349	1	0.07992
Amino sugar and nucleotide sugar metabolism	37	1	0.50708	1	0.12007

1) Total, the total number of compounds in the pathway.

2) Hits, the actually matched number from the user uploaded data.

3) Raw p, the original p value calculated from the enrichment analysis.

4) Holm adjust, p value adjusted by Holm-Bonferroni method.

5) Impact, the pathway impact value calculated from pathway topology analysis.

## SUPPLEMENTARY MATERIAL

**Supplementary Table S1**. Principal component analysis (PCA) and orthogonal partial least squares discriminant analysis (OPLS-DA) of goat semen

**Supplementary Table S2**. Differential metabolites of goat sperm between sodium salicylate group and control group

**Supplementary Table S3**. Enrichment analysis of differential metabolites in goat sperm from sodium salicylate group and control group

**Supplementary Table S4**. Analysis of the metabolic pathway of goat sperm in sodium salicylate group and control group

**Supplementary Figure S1**. Hierarchical cluster analysis of different metabolites in goat sperm between control group and sodium salicylate (SS) group.

**Supplementary Figure S2**. Correlation analysis of different metabolites in goat sperm between control group and sodium salicylate (SS) group.

## References

[1] Alvarenga MA, Papa FO, Neto CR (2016). Advances in stallion semen cryopreservation. Vet Clin North Am Equine Pract.

[2] Yanez-Ortiz I, Catalan J, Rodriguez-Gil JE, Miro J, Yeste M (2022). Advances in sperm cryopreservation in farm animals: Cattle, horse, pig and sheep. Anim Reprod Sci.

[3] Li Q, Weike S, Li Y (2018). Effects of oligomeric proanthocyanidins on quality of boar semen during liquid preservation at 17 degrees C. Anim Reprod Sci.

[4] Shaoyong W, Li Q, Ren Z (2019). Effects of kojic acid on boar sperm quality and anti-bacterial activity during liquid preservation at 17C. Theriogenology.

[5] Tian X, Li D, He Y (2019). Supplementation of salvianic acid A to boar semen extender to improve seminal quality and antioxidant capacity. Anim Sci J.

[6] Madunic J, Horvat L, Majstorovic I (2017). Sodium salicylate inhibits urokinase activity in MDA MB-231 breast cancer cells. Clin Breast Cancer.

[7] Odebiyi DO, Adigun OT, Kehinde MO (2007). Effect of sodium salicylate iontophoresis in the management of hip pain in patients with sickle cell disease. Nig Q J Hosp Med.

[8] Kazama I, Maruyama Y, Takahashi S, Kokumai T (2013). Amphipaths differentially modulate membrane surface deformation in rat peritoneal mast cells during exocytosis. Cell Physiol Biochem.

[9] Eidan SM (2016). Effect on post-cryopreserved semen characteristics of Holstein bulls of adding combinations of vitamin C and either catalase or reduced glutathione to Tris extender. Anim Reprod Sci.

[10] Hardie DG, Ross FA, Hawley SA (2012). AMP-activated protein kinase: a target for drugs both ancient and modern. Chem Biol.

[11] Feng TY, Lv DL, Zhang X (2020). Rosmarinic acid improves boar sperm quality, antioxidant capacity and energy metabolism at 17 degrees C via AMPK activation. Reprod Domest Anim.

[12] Martin-Hidalgo D, Hurtado de Llera A, Calle-Guisado V (2018). AMPK function in mammalian spermatozoa. Int J Mol Sci.

[13] Hawley SA, Fullerton MD, Ross FA (2012). The ancient drug salicylate directly activates AMP-activated protein kinase. Science.

[14] Dasgupta B, Seibel W, Neumann D, Viollet B (2018). Compound C/Dorsomorphin: its use and misuse as an AMPK inhibitor. AMPK. Methods in Molecular Biology.

[15] Vasquez J, Florentini EA, Camargo LA, Gonzales J, Valdivia M (2013). Hypoosmotic swelling test in epididymal ram (Ovis aries) spermatozoa. Livest Sci.

[16] Selvaraju S, Ramya L, Parthipan S (2021). Deciphering the complexity of sperm transcriptome reveals genes governing functional membrane and acrosome integrities potentially influence fertility. Cell Tissue Res.

[17] Ren F, Fang Q, Feng T (2019). Lycium barbarum and Laminaria japonica polysaccharides improve Cashmere goat sperm quality and fertility rate after cryopreservation. Theriogenology.

[18] Xu D, Wu L, Yang L (2020). Rutin protects boar sperm from cryodamage via enhancing the antioxidative defense. Anim Sci J.

[19] Karaji RO, Kia HD, Ashrafi I (2014). Effects of in combination antioxidant supplementation on microscopic and oxidative parameters of freeze-thaw bull sperm. Cell Tissue Bank.

[20] Uribe P, Villegas JV, Boguen R (2017). Use of the fluorescent dye tetramethylrhodamine methyl ester perchlorate for mitochondrial membrane potential assessment in human spermatozoa. Andrologia.

[21] Zhu Z, Kawai T, Umehara T (2019). Negative effects of ROS generated during linear sperm motility on gene expression and ATP generation in boar sperm mitochondria. Free Radic Biol Med.

[22] Lesich KA, Kelsch CB, Ponichter KL (2012). The calcium response of mouse sperm flagella: role of calcium ions in the regulation of dynein activity. Biol Reprod.

[23] El-Seadawy IE, Kotp MS, El-Maaty AMA, Fadl AM, El-Sherbiny HR, Abdelnaby EA (2022). The impact of varying doses of moringa leaf methanolic extract supplementation in the cryopreservation media on sperm quality, oxidants, and antioxidant capacity of frozen-thawed ram sperm. Trop Anim Health Prod.

[24] Ai J, Wu Q, Battino M, Bai W, Tian L (2021). Using untargeted metabolomics to profile the changes in roselle (Hibiscus sabdariffa L.) anthocyanins during wine fermentation. Food Chem.

[25] Li C, Ren C, Chen Y (2023). Changes on proteomic and metabolomic profiling of cryopreserved sperm effected by melatonin. J Proteomics.

[26] Akbari E, Beheshti F, Zarmehri HA, Mousavi SY, Gholami M, Ahmadi-Soleimani SM (2023). Comparative investigation of analgesic tolerance to taurine, sodium salicylate and morphine: involvement of peripheral muscarinic receptors. Neurosci Lett.

[27] Koga T, Kawata T (1989). Effects of food preservatives and local anesthetics on synthesis of outer membrane proteins in Vibrio parahaemolyticus. Tokushima J Exp Med.

[28] Marson FG (1954). Sodium salicylate and probenecid in the treatment of chronic gout; assessment of their relative effects in lowering serum uric acid levels. Ann Rheum Dis.

[29] Tsai TY, Lou SL, Wong KL (2015). Suppression of Ca2+ influx in endotoxin-treated mouse cerebral cortex endothelial bEND.3 cells. Eur J Pharmacol.

[30] Lim SC, Kim SM, Choi JE (2008). Sodium salicylate switches glucose depletion-induced necrosis to autophagy and inhibits high mobility group box protein 1 release in A549 lung adenocarcinoma cells. Oncol Rep.

[31] Bao W, Luo Y, Wang D, Li J, Wu X, Mei W (2018). Sodium salicylate modulates inflammatory responses through AMP-activated protein kinase activation in LPS-stimulated THP-1 cells. J Cell Biochem.

[32] Li RN, Zhu ZD, Zheng Y (2020). Metformin improves boar sperm quality via 5′-AMP-activated protein kinase-mediated energy metabolism in vitro. Zool Res.

[33] Hurtado de Llera A, Martin-Hidalgo D, Gil MC, Garcia-Marin LJ, Bragado MJ (2015). AMPK up-activation reduces motility and regulates other functions of boar spermatozoa. Mol Hum Reprod.

[34] Zhu Z, Li R, Fan X (2019). Resveratrol improves boar sperm quality via 5′ AMP-activated protein kinase activation during cryopreservation. Oxid Med Cell Longev.

[35] Wang W, Ye SD, Zhou KQ, Wu LM, Huang YN (2012). High doses of salicylate and aspirin are inhibitory on acid-sensing ion channels and protective against acidosis-induced neuronal injury in the rat cortical neuron. J Neurosci Res.

[36] Khandelia H, Witzke S, Mouritsen OG (2010). Interaction of salicylate and a terpenoid plant extract with model membranes: reconciling experiments and simulations. Biophys J.

[37] Gleitsman KR, Tateyama M, Kubo Y (2009). Structural rearrangements of the motor protein prestin revealed by fluorescence resonance energy transfer. Am J Physiol Cell Physiol.

[38] Hopp AK, Gruter P, Hottiger MO (2019). Regulation of glucose metabolism by NAD(+) and ADP-ribosylation. Cells.

[39] Ma D, Hu L, Wang J (2022). Nicotinamide mononucleotide improves spermatogenic function in streptozotocin-induced diabetic mice via modulating the glycolysis pathway. Acta Biochim Biophys Sin.

[40] Torres MA, Pedrosa AC, Novais FJ (2022). Metabolomic signature of spermatozoa established during holding time is responsible for differences in boar sperm freezability. Biol Reprod.

