# Supplementary material for: Antioxidant activity and metabolic regulation of sodium salicylate on goat sperm at low temperature
Source: Anim Biosci. 2024 Jan 20;37(4):640–54. doi: 10.5713/ab.23.0329 (PMC10915220; doi:10.5713/ab.23.0329)
Supplement: Supplementary file 1 [file ab-23-0329-Supplementary-Table-S1.pdf]

Table S1. Principal component analysis (PCA) and orthogonal partial least squares discriminant analysis (OPLS-DA) of goat semen.

Type	R^2X(cum)[1)]	R^2Y(cum)[2)]	Q^2(cum)[3)]
PCA	0.564		
OPLS-DA	0.455	0.958	0.581

[1), 2)] R^2X(cum) and R^2Y(cum): the cumulative explanatory power of variables.

[3)] Q^2(cum): the predictability of the model.
