# Supplementary material for: Antioxidant activity and metabolic regulation of sodium salicylate on goat sperm at low temperature
Source: Anim Biosci. 2024 Jan 20;37(4):640–54. doi: 10.5713/ab.23.0329 (PMC10915220; doi:10.5713/ab.23.0329)
Supplement: Supplementary file 2 [file ab-23-0329-Supplementary-Table-S2.pdf]

Table S2. Differential metabolites of goat sperm between sodium salicylate group and control group.

Name	rt[1)]	Mz[2)]	VIP[3)]	P-Value[4)]	FC[5)]
Cytosine	144.885	112.049751	1.64537307	0.03248472	0.74847443
Gentisaldehyde	20.4836	137.024932	2.63845026	5.146E-09	0.00872104
Adenosine	88.7628	326.112063	1.71309806	0.01090553	0.76948039
Adenosine monophosphate	249.379	348.067609	2.80430106	0.00011868	2.82272745
Allopurinol-1-ribonucleoside	129.574	269.086657	2.76234937	3.1512E-06	0.69859068
L-Malic acid	237.155	133.014766	2.63804102	4.2514E-05	0.69557517
L-Palmitoylcarnitine	103.043	400.339049	1.80122912	0.0226206	1.14987456
Propionylcarnitine	171.971	218.13747	2.46546902	0.00029639	1.30340093
Indolelactic acid	62.9337	204.067583	2.33077949	0.00024651	0.74347836
Salviaflaside methyl ester	130.129	537.164086	2.83376669	3.8618E-07	0.49873736
Hydroxyphenyllactic acid	106.166	181.051268	2.32138416	0.0002619	0.76467589
2',4'-Dihydroxyacetophenone	117.241	151.040632	1.95280197	0.00934029	1.44374684
Succinic anhydride	222.478	99.009182	1.87207676	0.03418053	0.91726108
N2-gamma-Glutamylglutamine	250.243	276.117127	2.3107975	0.00196846	1.23336912
4-Hydroxyphenylpyruvate	40.439	179.035394	2.06098167	0.00639477	0.8101155
SM(d18:1/12:0)	116.953	647.509384	2.03911873	0.02038778	0.84572597
Decanoylcarnitine	119.621	316.246038	1.74528271	0.02433946	1.29535336
2-Methylbenzoic acid	54.1835	135.045578	2.48880606	0.00118845	1.96547249
Phenyllactic acid	41.6306	165.05619	1.89660482	0.01180243	0.82482354

Butyrylcarnitine	159.834	232.152939	2.37800936	0.00252027	1.19234168
Dihydroxyacetone phosphate	250.769	168.991321	2.24914092	0.00038308	0.75628212
Dodecanoylcarnitine	113.583	344.277722	2.50032536	9.574E-05	1.21341971
2-Methylbutyroylcarnitine	150.074	246.168345	1.79901292	0.02096517	1.18311361
PE(16:0/18:2(9Z,12Z))	48.821	714.508096	2.32760168	0.00458889	0.80512985
NAD	252.884	664.113105	2.52639444	3.3699E-05	1.59446757
Ornithine	296.732	133.096207	2.13126762	0.02155436	1.96044336
Bergapten	153.457	215.033764	2.09432555	0.0129172	0.9412217
Byssochlamic acid	41.096	331.120379	2.05902595	0.00356578	0.62174216
3-Hydroxycinnamic acid	106.256	163.040634	2.17687818	0.00122621	0.78572569
SM(d18:1/14:0)	115.565	675.539984	1.9935302	0.02086708	0.8657293
Sphingosine	32.752	300.287503	1.75547405	0.04379473	0.89164425
PE(20:0/14:1(9Z))	50.4916	718.534102	1.68289486	0.04308419	0.83886751
PC(18:0/14:0)	154.3485	734.564016	1.36220621	0.04360879	0.73634648
Cytidine	144.921	244.091351	1.87513579	0.01460299	0.74908347
5-(10,13-Nonadecadienyl)-1,3-benzenediol	108.018	373.311235	2.36351277	0.00165198	1.21065906
(6beta,22E)-6-Hydroxystigmasta-4,22-dien-3-one	98.7348	427.357736	1.63721518	0.0396049	1.17569955
Epidermin	235.382	262.126563	2.90885095	3.7451E-07	2.02615141
Linoelaidyl carnitine	36.21755	424.339013	2.13736007	0.00604428	1.66141622
Hydrocortisone	151.837	407.204619	1.9766737	0.00812477	0.79439652

Nicotinamide ribotide	266.69	335.062063	2.10258678	0.00239338	0.69960947
(1S,2S,4R,8R)-p-Menthane-1,2,9-triol	26.8294	189.147003	1.47148404	0.04185157	1.38075874
alpha-Micropteroxanthin B	106.274	397.310989	1.6208913	0.03836713	1.22343478
L-Octanoylcarnitine	128.894	288.214717	2.38862406	0.00371981	1.5380465
PE(20:2(11Z,14Z)/14:0)	48.6721	716.515319	1.81281971	0.04609128	0.87256118
Panaquinquecol 4	207.417	275.158686	1.37030092	0.04988545	0.86632098
o-Cresol	40.4397	107.05052	2.07375375	0.00519492	0.78341281
7-Methylxanthine	153.904	165.04091	1.67237368	0.04445319	0.85330378
Stearoylcarnitine	98.6984	428.37116	2.00533358	0.00133101	1.21318417
cis-5-Tetradecenoylcarnitine	107.575	370.292822	2.61025063	0.00020875	1.2150617
Tryptophyl-Proline	155.2235	302.147927	1.90963519	0.01378864	0.77735501
PE(18:0/14:1(9Z))	51.609	690.50073	1.97643522	0.01577933	0.7863388
PC(16:0/15:0)	50.6692	720.538132	1.71976415	0.03906003	0.81980463
2-Oxo-4-methylthiobutanoic acid	31.1015	147.012488	2.15834305	0.00358586	0.67414151
25-Hydroxycholesterol	101.235	425.342195	1.53362469	0.04528493	1.19382746
Phosphatidylserine	227.3895	386.121524	1.20589056	0.04933785	1.25381893
Oxymorphone	245.491	302.132378	1.52399959	0.0264967	1.12336723
5'-Inosinic acid	359.823	347.03964	2.11569947	0.00929454	0.88299891
PC(20:2(11Z,14Z)/15:0)	42.1996	772.575503	1.76189692	0.01518659	0.75001967
Calcitriol	103.468	399.326585	1.55770521	0.04545837	1.15471717
PE(22:1(13Z)/14:1(9Z))	46.6432	744.546184	1.85386782	0.02207349	0.86045826
3-Methyl-2-oxopentanoate	28.4272	129.056205	2.34728191	0.00063115	0.75204685

N-Acetyl-D-glucosamine	215.884	220.083652	1.62818647	0.03929164	0.81045619
trans-Hexadec-2-enoyl carnitine	103.5	398.324472	1.52631875	0.0478338	1.15014666
N-Nitroso-pyrrolidine	11.4462	101.070247	1.39363499	0.03993498	0.7938831
(25S)-26-Hydroxy-24-methylenecycloartan-3-one	94.69145	455.390393	2.67696333	8.1262E-05	2.63481592
2-Acetylpyrazine	31.001	123.054497	1.72616136	0.01934844	0.88505049
4-(4-Hydroxyphenyl)-2-butanone glucoside	130.129	327.139479	2.79179634	4.535E-06	0.65597281
trans-Cinnamic acid	41.6919	147.045635	1.96254188	0.00605435	0.80441823
4-(Methylthio)-2-butanol	96.26625	121.068123	1.07410889	0.04976974	1.71975925
1-Isothiocyanato-4-phenylbutane	206.679	192.085252	1.76714009	0.01743738	0.76315276
Docosa-4,7,10,13,16-pentaenoyl carnitine	91.1821	474.355532	1.88375616	0.0134495	1.13155768
Piceatannol 4'-galloylglucoside	130.89	559.148237	2.32546761	0.00059374	0.64317509
LysoPC(22:6(4Z,7Z,10Z,13Z,16Z,19Z))	116.7145	568.337445	1.9484692	0.014815	0.77888718
L-Hexanoylcarnitine	141.314	260.183956	2.43026845	0.00195997	1.60700654
Picrotin	88.7628	309.096693	1.81640061	0.00783319	0.74368241

[1)]rt: the chromatographic retention time of the substance.

[2)]mz: the mass to charge ratio of characteristic ions in a substance.
[3)]VIP: variable importance in projection.
[4)]P-Value: obtained from the t-test of the substance in this group comparison.
[5)]FC: fold change.
