# Supplementary material for: Antioxidant activity and metabolic regulation of sodium salicylate on goat sperm at low temperature
Source: Anim Biosci. 2024 Jan 20;37(4):640–54. doi: 10.5713/ab.23.0329 (PMC10915220; doi:10.5713/ab.23.0329)
Supplement: Supplementary file 3 [file ab-23-0329-Supplementary-Table-S3.pdf]

Table S3. Enrichment analysis of differential metabolites in goat sperm from sodium salicylate group and control group.

Name	Number	P-Value[1)]	Name	Number	P-Value
Metabolic pathways	142	0.03920412	Cysteine and methionine metabolism	9	0.29831234
Glycerophospholipid metabolism	11	0.00018530	Arginine and proline metabolism	12	0.32073586
ABC transporters	32	0.00546160	Phenylalanine metabolism	4	0.23086847
Sphingolipid signaling pathway	4	0.00005720	Glutathione metabolism	6	0.18397061
Purine metabolism	9	0.09603399	Amino sugar and nucleotide sugar metabolism	5	0.47127197
Pyrimidine metabolism	9	0.04460197	Glycerolipid metabolism	4	0.18397061
D-Amino acid metabolism	20	0.04845030	Inositol phosphate metabolism	2	0.22253793
Sphingolipid metabolism	5	0.00752160	Glycosylphosphatidylinositol (GPI)-anchor biosynthesis	1	0.0210905
Nicotinate and nicotinamide metabolism	8	0.03381253	Arachidonic acid metabolism	5	0.33168896
Carbon metabolism	14	0.12162100	Linoleic acid metabolism	4	0.13897276

2-Oxocarboxylic acid metabolism	18	0.15803093	alpha-Linolenic acid metabolism	1	0.20988011
Biosynthesis of amino acids	25	0.14687691	Pyruvate metabolism	3	0.15724957
Biosynthesis of cofactors	25	0.52938525	Glyoxylate and dicarboxylate metabolism	9	0.28297182
cGMP-PKG signaling pathway	2	0.00118424	Propanoate metabolism	7	0.1926961
cAMP signaling pathway	6	0.00752160	Thiamine metabolism	5	0.15271547
Neuroactive ligand-receptor interaction	7	0.03048344	Drug metabolism - other enzymes	2	0.24320438
AMPK signaling pathway	3	0.00584793	Fatty acid metabolism	2	0.48273313
Longevity regulating pathway	2	0.00074164	Biosynthesis of nucleotide sugars	5	0.66404274
Necroptosis	3	0.00118424	Antifolate resistance	3	0.08673585
Retrograde endocannabinoid signaling	5	0.00437110	FoxO signaling pathway	2	0.02629626
Taste transduction	8	0.01215850	Autophagy - other	1	0.01585813

Regulation of lipolysis in adipocytes	5	0.00236401	Autophagy - animal	1	0.03147554
Renin secretion	3	0.00349895	mTOR signaling pathway	3	0.0210905
Aldosterone synthesis and secretion	4	0.00584793	PI3K-Akt signaling pathway	1	0.0210905
Cortisol synthesis and secretion	2	0.00172568	Apoptosis	1	0.0210905
Parathyroid hormone synthesis, secretion and action	2	0.00118424	Vascular smooth muscle contraction	2	0.08183956
Cushing syndrome	2	0.00203286	Olfactory transduction	1	0.04175515
Parkinson disease	5	0.00812218	Glucagon signaling pathway	5	0.12969278
Morphine addiction	2	0.00074164	Alcoholic liver disease	2	0.0569791
Pathways in cancer	4	0.01143533	Aldosterone-regulated sodium reabsorption	1	0.04175515
Choline metabolism in cancer	6	0.00144272	Endocrine and other factor-regulated calcium reabsorption	1	0.04175515
Glycolysis / Gluconeogenesis	4	0.15271547	Proximal tubule bicarbonate reclamation	3	0.08673585

Citrate cycle (TCA cycle)	4	0.10127531	Bile secretion	9	0.40700384
Pentose and glucuronate interconversions	7	0.26731081	Vitamin digestion and absorption	4	0.18834457
Fructose and mannose metabolism	2	0.25132293	Mineral absorption	10	0.14357718
Galactose metabolism	4	0.21834037	Pathways of neurodegeneration - multiple diseases	4	0.15724957
Fatty acid degradation	3	0.23500166	Alcoholism	4	0.05193034
Steroid biosynthesis	1	0.26731081	Tuberculosis	1	0.04685573
Primary bile acid biosynthesis	4	0.22253793	Kaposi sarcoma-associated herpesvirus infection	1	0.02629626
Ubiquinone and other terpenoid-quinone biosynthesis	3	0.31704682	Chemical carcinogenesis - reactive oxygen species	5	0.26334474
Steroid hormone biosynthesis	2	0.41343270	Renal cell carcinoma	1	0.01585813

Oxidative phosphorylation	2	0.08183956	Prostate cancer	1	0.0569791
Arginine biosynthesis	7	0.11559321	Central carbon metabolism in cancer	18	0.1795741
Caffeine metabolism	3	0.11084500			

[1)]P-Value: obtained from the t-test of the substance in this group comparison.
