# Supplementary material for: Antioxidant activity and metabolic regulation of sodium salicylate on goat sperm at low temperature
Source: Anim Biosci. 2024 Jan 20;37(4):640–54. doi: 10.5713/ab.23.0329 (PMC10915220; doi:10.5713/ab.23.0329)
Supplement: Supplementary file 4 [file ab-23-0329-Supplementary-Table-S4.pdf]

Table S4. Analysis of the metabolic pathway of goat sperm in sodium salicylate group and control group.

Pathway	Total[1)]	Hits[2)]	Raw p[3)]	Holm adjust[4)]	Impact[5)]
Caffeine metabolism	12	1	0.20334	1	0
Nicotinate and nicotinamide metabolism	13	1	0.21836	1	0.2439
Glycerolipid metabolism	18	1	0.28947	1	0
Fructose and mannose metabolism	19	1	0.30293	1	0.02273
Sphingolipid metabolism	21	1	0.32911	1	0.05263
Purine metabolism	68	2	0.36603	1	0.05954
Glycolysis or Gluconeogenesis	26	1	0.39048	1	0
Glutathione metabolism	26	1	0.39048	1	0
Inositol phosphate metabolism	28	1	0.41349	1	0
Cysteine and methionine metabolism	28	1	0.41349	1	0.07992
Glycerophospholipid metabolism	29	1	0.42468	1	0.02442
Steroid biosynthesis	35	1	0.48762	1	0
Pyrimidine metabolism	37	1	0.50708	1	0.01135
Amino sugar and nucleotide sugar metabolism	37	1	0.50708	1	0.12007
Fatty acid metabolism	39	1	0.52584	1	0
Arginine and proline metabolism	44	1	0.56977	1	0.12445
Primary bile acid biosynthesis	46	1	0.58623	1	0.01057

[1)]Total: the total number of compounds in the pathway.

[2)]Hits: the actually matched number from the user uploaded data.

[3)]Raw p: the original p value calculated from the enrichment analysis.

[4)]Holm adjust: p value adjusted by Holm-Bonferroni method.

[5)]Impact: the pathway impact value calculated from pathway topology analysis.
