# Supplementary material for: Antioxidant activity and metabolic regulation of sodium salicylate on goat sperm at low temperature
Source: Anim Biosci. 2024 Jan 20;37(4):640–54. doi: 10.5713/ab.23.0329 (PMC10915220; doi:10.5713/ab.23.0329)
Supplement: Supplementary file 6 [file ab-23-0329-Supplementary-Fig-S2.pdf]

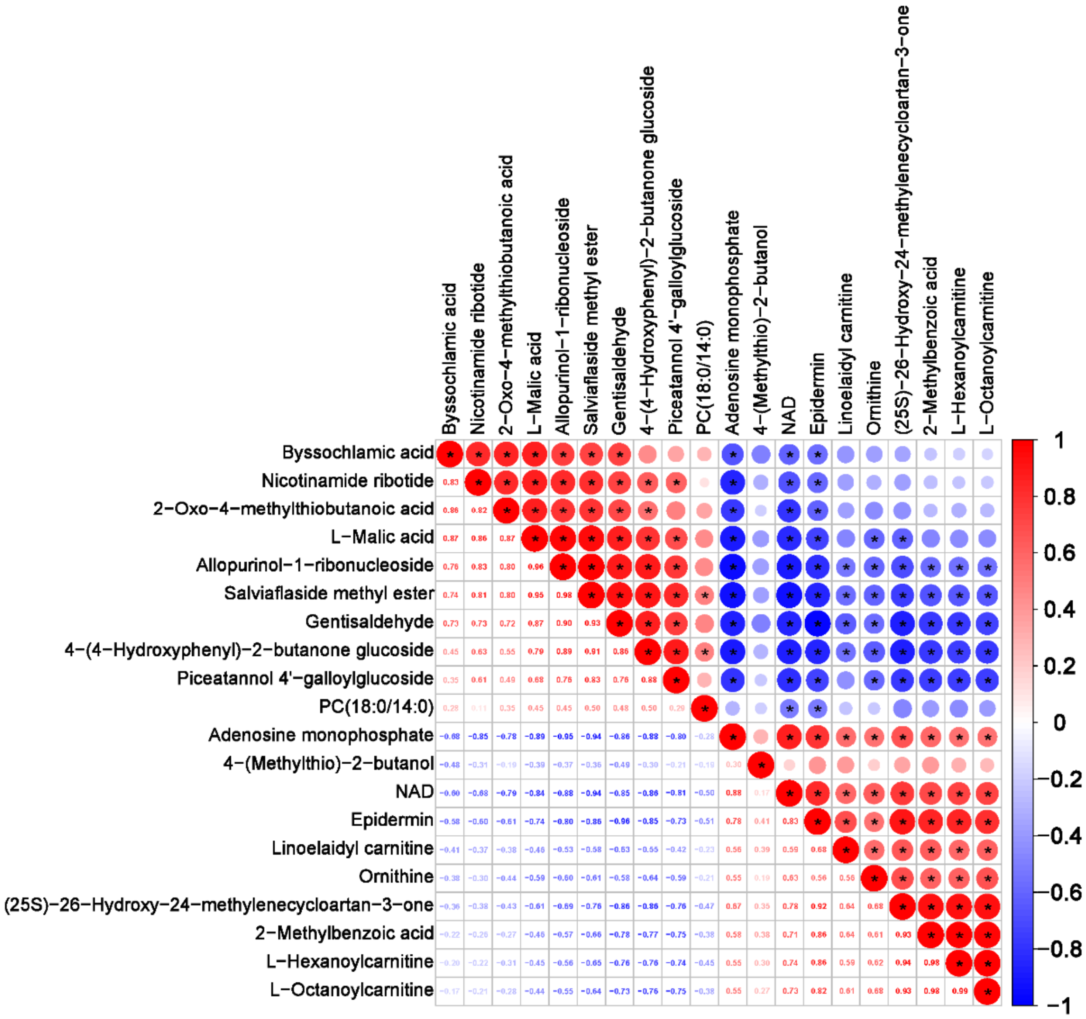

Figure S2. Correlation analysis of different metabolites in goat sperm between control group and sodium salicylate (SS) group. * means significant. r is the correlation coefficient, and the closer the absolute value of r is to 1, the stronger the correlation between the two variables.
